# Combining early lower eyelid surgery with neuromuscular retraining for synkinesis prevention after facial palsy: the role of the eye in aberrant facial nerve regeneration

**DOI:** 10.3389/fneur.2024.1443591

**Published:** 2024-09-18

**Authors:** Arianna Di Stadio, Massimo Ralli, Pietro De Luca, Jake Sossamon, Teresa C. Frohman, Marta Altieri, Ignazio La Mantia, Salvatore Ferlito, Elliot M. Frohman, Michael J. Brenner

**Affiliations:** ^1^Otolaryngology Unit, Department GF Ingrassia, University of Catania, Catania, Italy; ^2^Organ of Sense Department, University La Sapienza, Rome, Italy; ^3^Otolaryngology Department, Fatebenefratelli-Isola Hospital, Rome, Italy; ^4^Medical University of South Carolina, Charleston, SC, United States; ^5^Distinguished Senior Fellows (Sabbatical) Neuroimmunology Laboratory of Professor Lawrence Steinman, Stanford University School of Medicine, Palo Alto, CA, United States; ^6^Neurology Department, University La Sapienza, Rome, Italy; ^7^Department of Otolaryngology—Head and Neck Surgery, University of Michigan Medical School, Ann Arbor, MI, United States

**Keywords:** facial paralysis, synkinesis, paralytic ectropion, lagophthalmos, oculoplastic surgery, aberrant facial reinnervation syndrome

## Abstract

**Background:**

Facial synkinesis (FS) is a distressing sequela of facial palsy (FP) characterized by involuntary, simultaneous movements of facial muscles occurring during voluntary facial expressions. Treatment of synkinesis is challenging, and preventive methods are needed.

**Aim:**

This study evaluated the efficacy of physical facial nerve rehabilitation (PFNR) therapy alone vs. PNFR with eyelid surgery to correct lagophthalmos and prevent the onset of synkinesis.

**Methods:**

Twenty five outpatients were randomized to receive either PFNR alone (neuromuscular retraining and Kabat proprioceptive neuromuscular facilitation) or PNFR and early (90 days after FP onset) eyelid surgery (involving a conservative oculoplastic correction for lagophthalmos with epiphora or ectropion). Comprehensive otolaryngological assessments and Magnetic Resonance Imaging (MRI) were conducted. Synkinesis progression was measured using Another Disease Scale (ADS) at baseline, 3-, 6-, 12-, and 24-months post-treatment. The data were analyzed with ANOVA, *τ*-test, Chi-Square analyses.

**Results:**

Patients undergoing eyelid surgery with PFNR showed faster (*p* < 0.001) and better recovery of facial movements (*p* < 0.05) than patients receiving PFNR alone comparing T0 and T12 (*p* < 0.0001). No synkinesis were observed in the PFNR plus surgery group while 37% of patients in PFNR alone had synkinesis (*p* = 0.03). At 24 months, none of the patients in the surgery group presented synkinesis.

**Conclusion:**

Combining early surgical treatment of paralytic lagophthalmos or epiphora with PFNR accelerated functional recovery and reduced synkinesis in patients with FP compared to facial rehabilitation alone. Further investigations in larger populations with long-term follow-up are needed.

**Clinical trial registration:**

https://clinicaltrials.gov/study/NCT06538103, NCT06538103.

## Introduction

Facial paralysis results in functional impairments that affect facial expression, eating, speaking, and eye closure, collectively impairing daily activities and quality of life. The disfigurement associated with facial paralysis can lead to altered self-perception, social stigma, withdrawal, depression, and anxiety. Bell’s palsy, the most common cause of facial paralysis, has an annual incidence of 23–53 per 100,000 individuals (1, 2). Facial paralysis ranges in severity and can involve dynamic and static function.

Synkinesis, a manifestation of aberrant facial nerve reinnervation syndrome, is a common and distressing sequela of facial paralysis. Most often affects eye closure (2) chewing and drinking, due to impairment of orbicularis oculi and oris muscles (3). Patients often adapt their eating habits to compensate for masticatory deficits (3). The disorder is characterized by involuntary simultaneous movement of different facial muscle groups and can be accompanied by abnormal facial tone and spasm; it occurs in up to 78% of patients during the recovery of facial movement (4). Synkinesis often develops after stroke-associated facial paralysis (5). It is attributed to misdirected regeneration of injured facial nerve fibers, aberrant muscle reinnervation, and cortical rearrangement (6). Oculo-oral synkinesis refers to synkinesis principally affecting the orbicularis muscles of the eyes and mouth (i.e., orbicularis oculi and the orbicularis oris muscles) (6).

We hypothesized that early intervention to prevent eyelid dysfunction might reduce patients’ risk of developing synkinesis. The eye secretes through the retina nerve growth factors (NGF) (7, 8), especially when cornea surface becomes dry. In facial paralysis, impaired eyelid function predisposes to inadequate eyelid closure (lagophthalmos), tearing (epiphora), and eyelid eversion (ectropion) (2, 9). Lack of eye protection can cause exposure keratitis, vision loss, and increased light exposure to the retina that alters retinal signaling. Current interventions for ocular synkinesis aim at symptomatic relief, rather than prevention. Temporary solutions like bandaging, gel drops and adhesive palpebral weight but provide interim relief until eyelid motility is recovered (10). Although Botulinum toxin affords temporary chemodenervation, synkinesis inevitably returns. Neurectomy or myectomy may allow for longer-term effects; but there are higher risk of complications and benefits are often temporary (6, 11).

Non-invasive strategies aiming to reorganize the facial motor cortex, such as neuromuscular retraining, Kabat technique (also known as Proprioceptive Neuromuscular Facilitation), or biofeedback require ongoing commitment and typically result in incomplete recovery (5, 12). Although preclinical studies have studied neuro-inhibition in preventing synkinesis (13), clinical studies on synkinesis prevention are lacking.

On the other side, several of oculoplastic interventions have been studied for eyelid dysfunction (14), including eyelid weights (15, 16), wedge excision (16), lateral canthoplasty (17), and other approaches (18) but nobody evaluated the effect of these surgeries on synkinesis onset, nor the combination of surgery with facial therapy to prevent this disfiguring condition. All such procedures have variable success in controlling symptoms and intermittent complications. All such procedures have variable success in controlling symptoms and intermittent complications (14–17). In addition, some of these surgical interventions offer poor esthetic results (16, 17).

Di Stadio proposed a minimally invasive technique to treat eyelid dysfunction (18), the main benefits of this technique are (i) being specific for the eye concern (different approaches for lagophthalmos, epiphora, or ectropion) and (ii) offering excellent esthetic results (18). This minimal eyelid surgery use musculocutaneous flap to support the eyelid and is less invasive than shortening the lower placing eyelid weights, tarsal plate repositioning, or canthopexy (18). This office-based eyelid surgery might be useful to treat deficit in eye closure, reducing the risk of corneal desiccation, and excessive retinal light exposure which might be implicated in the onset of synkinesis.

By the way, the effect of this technique as well as overmentioned other ones (14–17) has never been evaluated on synkinesis prevention, not alone nor associated to physical rehabilitation. Furthermore, it was not compared with traditional physical rehab as preventor of synkinesis.

This study aimed at investigating if minimally invasive eyelid surgery for patients with facial paralysis can reduce the onset of synkinesis. We therefore explored whether this early, pre-emptive eyelid surgery could improve the recovery trajectory of facial paralysis, alleviate risk of developing periorbital complications, and prevent the onset of synkinesis.

## Materials and methods

This study was conducted at a tertiary referral hospital from January 2022 to November 2023. Patients with facial paralysis (FP) were consecutively recruited from the otolaryngology department upon presentation. The hospital’s Internal Review Board (IRB) approved the study with number CT X06, which adhered to the Helsinki Declaration for human rights. The study was registered on Clinicaltrial.gov with identification number NCT06538103. After being informed about the study’s objectives and procedures, all participants provided written consent, which included permission to share their anonymized data for scientific purposes.

The otolaryngologist research team collected patient histories and data on age, sex, known systemic diseases, and family history of neurological conditions. A comprehensive otolaryngological examination was performed, including head and neck exam, microscope exam of bilateral ears, equilibrium testing, and facial nerve scoring using the Another Disease Scale (ADS) scale (9) and a validated new assessment. This assessment evaluates the movement of the facial muscles both analyzing the superior, middle, and inferior segment of the face singularly and the entire facial expression. Patients are invited to perform some expressions, i.e., smiling or kissing, so that physician can understand which muscle has a functional deficit. Moreover, the ADS scale integrates synkinesis into the scoring scheme and segments the face into upper, middle, and lower regions, facilitating a nuanced evaluation of facial paralysis and synkinesis. Computer tomography (CT) scan was performed to rule out stroke or other vascular pathology. An internal medicine consultation was also sought to corroborate cause of facial paralysis. Patients with systemic or neurological diseases potentially causing facial paralysis were referred to specialists and excluded from the study.

All patients in the study initiated facial nerve therapy (19, 20) and medical therapy as indicated. Individuals diagnosed with idiopathic facial paralysis (Bell’s palsy) received steroid therapy in accordance with clinical practice guidelines (21). Individuals diagnosed with Ramsay Hunt syndrome (herpes zoster oticus) received acyclovir 800 mg, prescribed five times daily for 10 days. Individuals with post-surgical (e.g., post-vestibular schwannoma resection) or stroke-related facial paralysis underwent only physical therapy and supportive care.

After completing the acute phase of care (10 days), the patients who presented a total facial paralysis (0–3 ADS scores) were included in the controlled randomization process that assigned even numbers of patients with infectious-origin FP (suspected or confirmed) and odd numbers to those with stroke or previous surgery.

Randomization was conducted using a computer-generated random number sequence to assign participants to either the intervention or control group in a 1:1 ratio. Enrolled participant completed all baseline assessments and were assigned to their respective group. This process was designed to minimize selection bias and ensure the comparability of groups at baseline.

All patients were blinded to group assignment to minimize any psychological influences (positive or negative) that might bias the results.

The sample size was calculated considering a 95% confidence interval (CI), a 5% margin of error, and a 50% population proportion, requiring 28 subjects for the original enrollment. The chosen sample size aims for a precise estimation within the 95% CI, acknowledging the potential for minimal margin of error. For this reason, we decided to include 30 patients. A computerized system allocated 30 participants 1:1 to one to the following treatment regimens resulting in 15 participants per group:

- Treatment Group 1 (TG1): Kabat therapy (22) and neuromuscular reeducation (NMR) at home (23).
- Treatment Group 2 (TG2): Kabat therapy (22), home NMR, and eyelid surgery at 90 days (starting the count from day one of treatment) (if lagophthalmos with epiphora or ectropion was present) (18).

Kabat therapy involved thrice-weekly 35-min sessions. NMR was done twice weekly for 15 min, using a mirror. Patients were assessed at 3, 6, 9, and 12 months. Those with persisting deficits at 12 months received monthly video-call follow-ups until full recovery. Physical therapy lasted at least 12 months, except for those who recovered earlier. The study team performed video-call check-ins every 3 months for a year post-therapy, to assess facial function, with a final follow-up at 24 months post-facial paralysis onset.

We used physical rehabilitation in both groups because this approach followed the standard treatment for facial palsy as described by Robinson et al. (23). For ethical reasons, it was not possible to include different control groups, as for example patients treated with eye surgery only or untreated patients.

**Figure 1:**
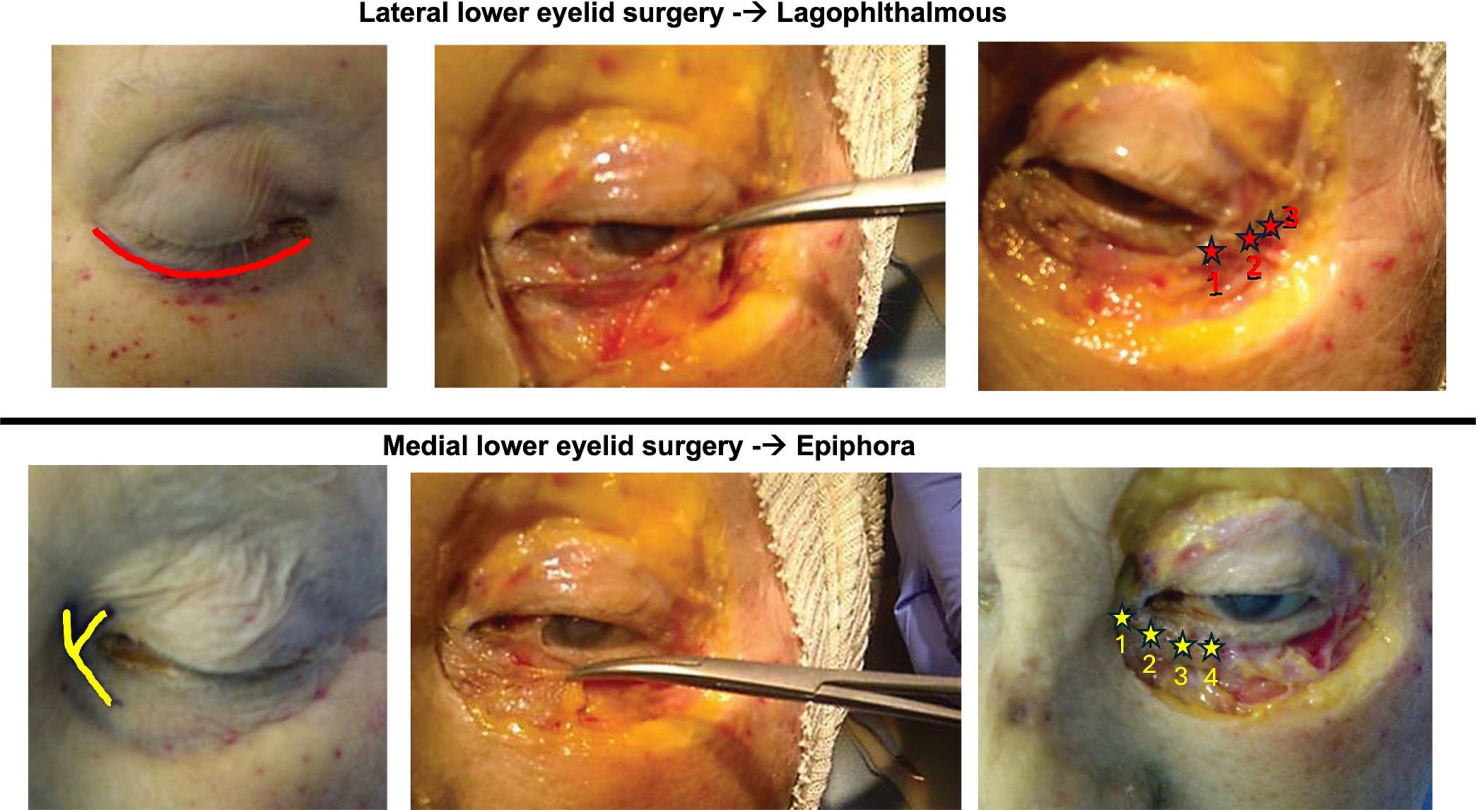
Image modified from Di Stadio (18). The upper row of images depicts the laterally based technique used for lagophthalmos. Using subciliary access incision, the surgeon elevates a lateral orbicularis muscle flap, taking care to preserve its neurovascular supply. The muscle flap is suspended laterally by anchoring it to the condensation of connective tissue comprising Whitnall’s tubercle. The lower row of images demonstrates the medially based lower eyelid surgery used for treating epiphora. Using a y-shaped incision, the surgeon elevates the medial orbicularis muscle, preserving neurovascular supply, and anchoring the flap to the connective tissue and medical canthal tendon with four sutures, allowing more efficient drainage (red stars).

Early lower eyelid surgery was performed in accordance with the protocol previously described by Di Stadio (18), with medial or lateral eyelid lifting surgery as indicated. The indication for eye surgery were in case of ectropion (exposure of the conjunctiva due to a reduction in tension of the anterior compartment of the eye muscle) and lagophthalmos (incomplete/abnormal closure of the eye with eyelid in closed position) a lateral lower eyelid lifting surgery was performed (18); otherwise, in presence of epiphora (the eversion of the lachrymal point) and lagophthalmos medial lower eyelid lifting surgery was done (18) (Figure 1). We used ocular surface area (OSA) measurement to evaluate the exposure of sclera (24).

To reduce operator dependent bias, the eye surgery was always performed by the same surgeon with over a decade of experience in facial plastic surgery (ADS).

The study incorporated safety provisions. Lower eyelid surgery was the only “at risk” procedure; inflammation and/infection of the sutures could happen rarely. To minimize this risk, all patients were treated for 6 days by antibiotic for os (Amoxicillin 1 g each 12 h) associated to eye drops (Tobramicin 0.3%, one drop three times a day). Patients also received standard postoperative follow-up.

Inclusion criteria were being over 18 years old, willingness to participate, and acute/new onset of FP (less than 15 days).

Exclusion criteria included neuro-inflammatory diseases (e.g., multiple sclerosis), FP from systemic disorders (e.g., Guillain-Barré syndrome, Lyme disease, and encephalitis), FP from middle ear infections or untreated cholesteatoma, severe cognitive or psychological disorders, or non-consent to participate in the study. Patients who did not complete at least two follow-ups were excluded from the final analysis and considered dropouts.

The primary outcomes of this study were to evaluate the incidence of the facial synkinesis and the final score at the end of the therapy (best scores correspond to best recovery).

The secondary outcome was to evaluate the time to recover normal facial function.

### Statistical analyses

We compared the changes of ADS scale scores within and between the two groups using One-way ANOVA and Bonferroni-Holmes (BH) *ad hoc* test at the baseline and at the end of the observation period (12 months). The differences in the months of recovery were analyzed by *τ*-test (τ). The comparison between the presence and not of synkinesis was done by using Chi-Square (*χ*) test. All analyses were performed using Stata ®. *p* was considered statistically significant <0.05.

## Results

### General

All patients included in the study were suffering from severe facial paralysis, 0–3 scores in according to ADS, without any movements. Of the 30 patients enrolled in the study, 25 (84%) completed at least two follow-ups, including the one at 12 months. These participants (11 male, 14 female) were age 46.5 ± 15.9 years (CI 95% 23–75). Of the 25 patients, 12 patients were diagnosed with Bell’ palsy, 12 presented with a facial paralysis after surgery, one patient had a stroke, and there were no cases of Ramsey Hunt syndrome or other etiologies. Of the post-surgical patients, nine had a facial paralysis as consequence of vestibular schwannoma removal, two due to surgical removal of a VII schwannoma, and one case had facial paralysis after timpano-jugular paraganglioma surgery. None of the patients who underwent surgery had facial nerve sacrifice or reconstruction. Fourteen patients were in the facial nerve therapy group with only one patient lost (6.6% drop) and 11 in surgery plus facial nerve therapy group (26.6% drop; four patients did not complete the follow-ups).

During the study, none of the patients received botulinum toxin or underwent other procedures related to their facial paralysis. All patients had evidence of superior eyelid movement after 12 weeks but at least some residual eyelid dysfunction, involving mild to moderate lagophthalmos with either ectropion, epiphora, or a combination of these symptoms. In the surgery plus facial nerve treatment group, there was improvement of symptoms in all cases. Table 1 summarizes the characteristics of treatment group 1 (facial nerve therapy alone); nine patients were affected by a right facial paralysis (64.3%) and five by a left sided paralysis (35.7%). Table 2 summarizes characteristics of treatment group 2 (surgery plus facial nerve therapy); seven patients suffered from a right facial paralysis (63.6%) and four from a left sided paralysis (36.4%).

**Table 1:** Demographic of TG1.

Patients	Sex	Age	ADS score T0	Side and cause	Synkinesis	Recovery time months	Final ADS score
6	m	75	2.3	Right; Bell’s	Yes	13	4.7
7	f	33	0	Right; Paraganglioma	Yes	12	5
8	m	22	2.7	Left; Bell’s	No	7	9
10	f	29	0	Right; Bell’s	No	16	3.1
11	f	25	1.8	Left; Vestibular Schwannoma	No	7	9
15	m	57	1.8	Right; Vestibular Schwannoma	Yes	14	5.6
16	f	45	1.8	Right; Bell’s	No	6	9
18	f	63	3	Right; Bell’s	No	11	8.1
19	f	60	0.9	Left; Vestibular Schwannoma	Yes	12	5.3
20	m	36	1.8	Right; Bell’s	No	11	8.7
21	f	47	3	Right; Vestibular Schwannoma	No	12	8.4
22	f	52	3	Left; Bell’s	Yes	14	5.2
23	m	51	2.7	Left; Facial Schwannoma	No	10	8.4
25	m	55	2.4	Right; Vestibular Schwannoma	No	11	8.4

Demographic characteristics of patients included in TG1 who performed only physical rehabilitation.

**Table 2:** Demographic of TG2.

Patients	Sex	Age	ADS score at T0	Side and cause	Synkinsis	Recovery time months from T0	Final ADS score
1	m	48	1.5	Right; Vestibular Schannoma	No	9	8.4
2	f	23	2.7	Left; Bell’s	No	8	8.7
3	f	40	0	Left; Vestibular Schannoma	No	8	8.7
4	m	62	1.2	Right; Bell’s	No	8	9
5	f	44	0	Left; Vestibular Schannoma	No	9	9
9	f	75	2.4	Right; Facial Schwannoma	No	7	9
12	f	20	1.5	Right Bell’s	No	8	8.7
13	m	35	0	Right;Vestibular Schannoma	No	7	9
14	m	47	0.3	Right; Bell’s	No	6	9
17	f	52	0	Right; Stroke	No	7	7.2
24	m	67	0.3	Left; Bell’s	No	7	9

Demographic characteristics of patients included in TG2 who performed physical rehabilitation with Kabat and eye surgery.

Two patients in the surgery plus facial nerve therapy group (TG2) had epiphora and lagophthalmos and were treated by medial lower eyelid surgery (n 9 and 24), the other nine were affected by ectropion and lagophthalmos for which lateral lower eyelid surgery was performed.

**Figure 2:**
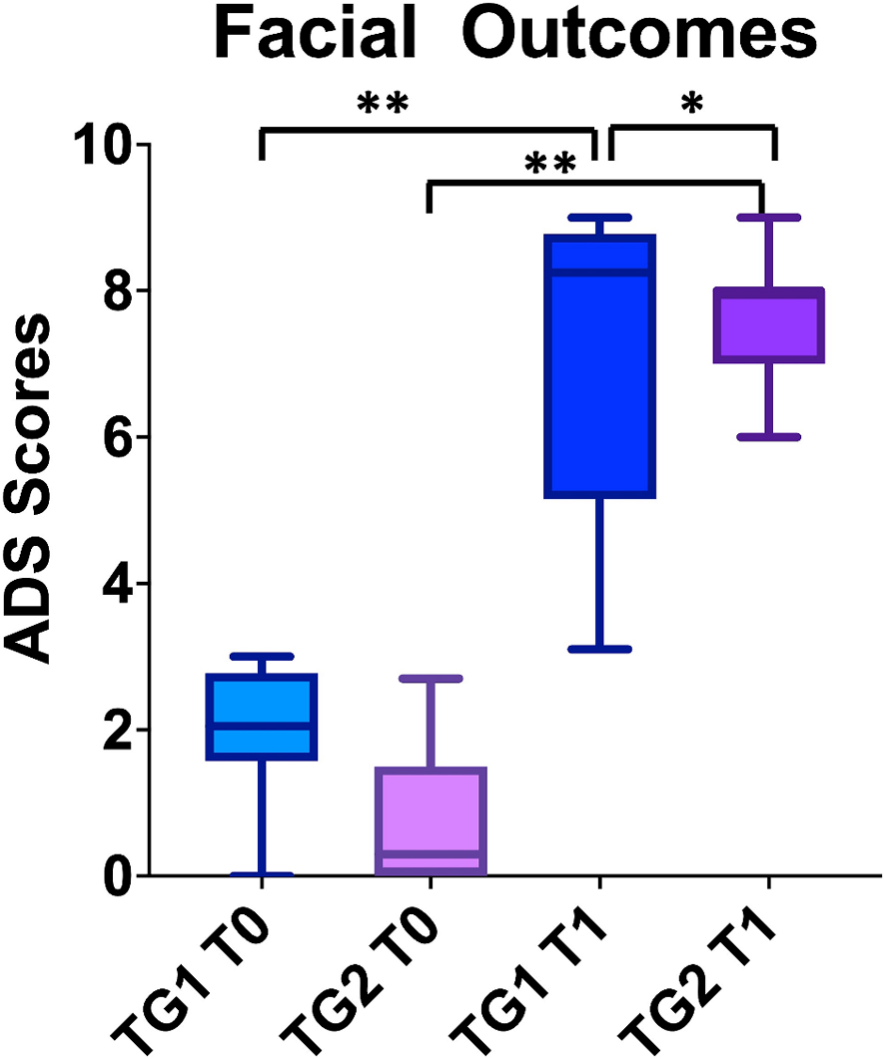
The graph bar compares the facial outcomes between the two groups at the baseline and at the end of the treatment. Higher the score better the facial motility. To note ADS scale considers synkinesis also, which presence decrease the final score. Despite both groups recovered from the baseline, the patients in TG2 group had better ADS scores. “*” *p* < 0.05, “**” *p* < 0.01.

All patients improved their ADS scores after treatment (ANOVA: *p* < 0.0001) both in facial nerve therapy alone (TG1; average 6.9 ± 2.4; CI95%: 3.1–9) (BH: *p* < 0.01) and surgery plus facial nerve therapy (TG2; average 8.7 ± 0.5; CI95%: 7.2–9) (BH: *p* < 0.01) (Figure 1). No statistically significant differences were observed between TG1 (average 1.9 ± 1; CI95%: 0–2.7) and TG2 (average 0.9 ± 1; CI95%: 0–2.7) before treatment (BH: *p* > 0.05). Statistically significant differences in term of ADS scores were identified after treatment comparing TG1 and TG2 (BH: *p* < 0.05); TG2 had higher ADS scores corresponding to better recovery (Figure 2).

**Figure 3:**
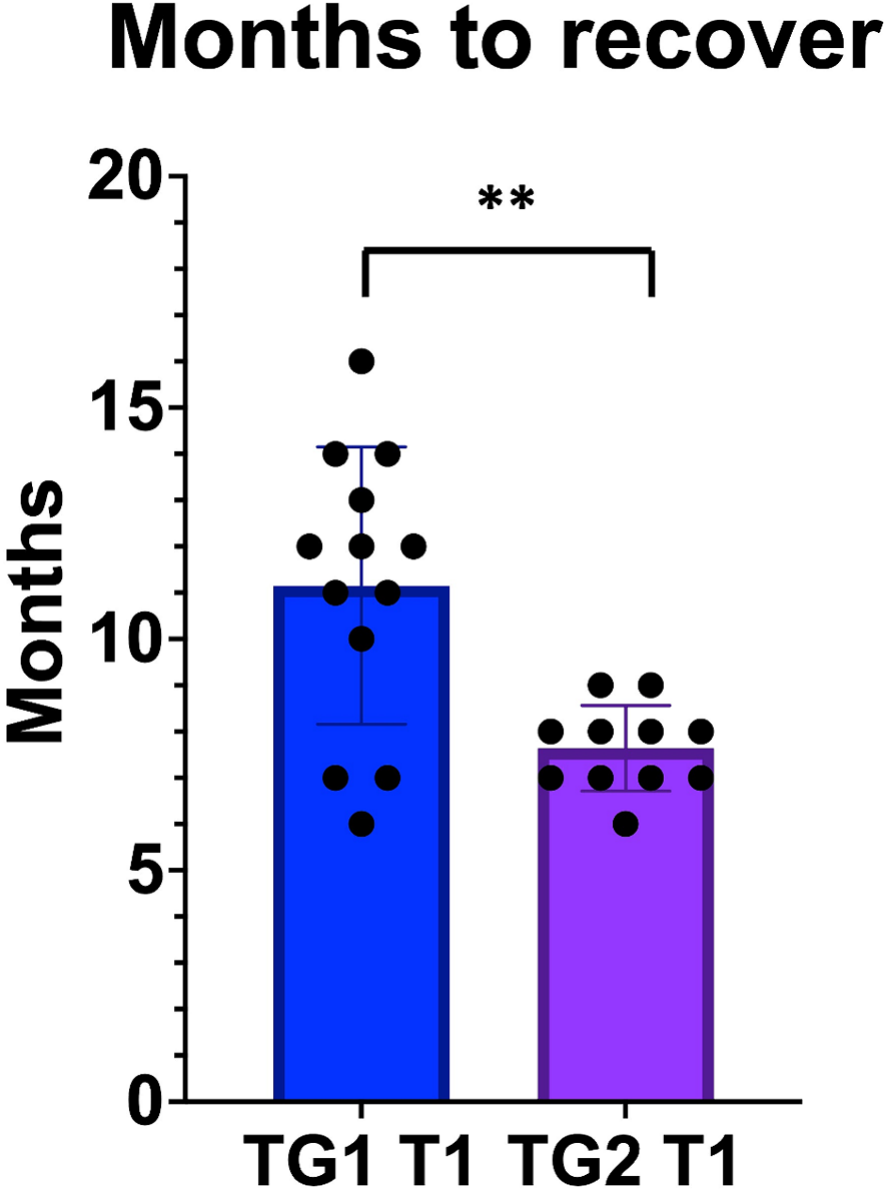
The patients in TG2, who underwent early eye surgery, recovered from FP earlier than TG1, treated by physical rehab only. “**” *p* < 0.01.

Statistically significant differences were observed between TG1 and TG2 at 3 months (*τ*: *p* < 0.001); TG2 recovered 11 ± 2.9 (average; CI95%: 6–13) and TG1 7.6 ± 0.9 (average; CI95%:7–9) (Figure 3). 100% of patients in TG2 stopped physical therapy before 12 months due to the recovery of their facial functions.

None of the patients in the surgery plus facial nerve therapy group had synkinesis after 12 months or 24 months from facial paralysis onset. In the facial nerve therapy alone group, five patients (35.7%) suffered from synkinesis on the side of the original paralysis, which was still present in all cases at the 24 months check. None of the other patients were affected by synkinesis at the last video-follow-up. The presence of synkinesis differed significantly between the two groups (*χ*: *p* = 0.03). The significant reduction in synkinesis in the surgery group thus supported a role for early eyelid surgery in enhancing facial nerve recovery outcomes.

## Discussion

Our study revealed that corrective eyelid surgery for paralytic lagophthalmos with facial nerve therapy was associated with shorter recovery period, reduced likelihood of developing synkinesis, and enhanced facial function compared to facial nerve therapy alone, based on ADS scores. Although a variety of oculoplastic interventions have been studied for eyelid dysfunction, including eyelid weights (16, 17), wedge excision (16), lateral canthoplasty (17), and other approaches (18), these interventions have not been systematically studied in combination with facial therapy or with specific emphasis on synkinesis. Furthermore, their role in preventing or alleviating synkinesis is unknown. The present study highlights a novel consideration in management of eyelid disorders with emphasis on early intervention providing eyelid suspensory anchoring and its potential role in mitigating synkinesis.

Regarding the use of eyelid surgery after 3 months from the onset of paralysis, it is important to underline that we only performed the surgery on patients with total absence of any facial movements. Anyway, it is also true that the timing to recover the movement can change between patients even depending on age (19–22) and in some cases (patients 2 and 12) the surgery could be delayed waiting for spontaneous recovery. The latter could also have impacted on the recovery especially in younger patients. On the other hand, retina of young people might be more sensitive to the light exposure than the one of older people (some of them can suffer from cataract) so the neuroepithelium of the structure might hyperproduces NGF (7, 8) causing an earlier onset of synkinesis than the one observed in aging people.

Some monkey’s studies should be performed to understand how long the surgery can be delayed at different ages. We suggested monkey because primates share 99% DNA with humans, and this could increase the accuracy of results avoiding controversies and doubts (25). However, because we only present a theoretic mechanism, preliminary studies using less expensive animals (mice and rats) might be considered. Once clarified this aspect, a tailor-made treatment could be suggested for each patient affected by FP.

Physical rehabilitation is a widely accepted treatment for facial paralysis as integral part of facial nerve rehabilitation and is often a first-line therapy for synkinesis (23). Various methods, including KABAT (19–21), Neuromuscular Training (NMR) (26), combinations thereof (27), and recently, KABAT with facial taping (28), have been described. In all studies, the use of facial rehabilitation was able to ameliorate the recovery in term of better facial motility and reduced time to recovery (21) independent from the primary cause of facial palsy (20, 21). In case of severe facial palsy, the benefit of rehabilitation was most apparent (19). Physical rehabilitation alone if using a single method (Kabat or NMR), a combination of methods, or associated with other technique commonly used in rehab (muscular taping) benefits patients when compared with control groups. Often, the eye is the portion of the face that tends to remain asymmetric or because of lack of complete closure or because of the onset of synkinesis (5). Despite the large success of physical rehabilitation on the restoration of facial motility, synkinesis sometimes occurs.

There are some cases, i.e., post angle ponto cerebellum schwannoma removal, in which physical rehabilitation cannot allow the recovery of facial movements causing invalidating sequela. In case of persistent facial palsy, there are several surgical procedures to use, surgical options include nerve transfers and free microvascular muscle transfer (29). However, these invasive procedures are complex (29) and to note, the long-term sequela, including late onset of synkinesis have never been investigated.

For less severe functional impairments, more typical of idiopathic facial paralysis or partial loss of function, a combination of minimally invasive techniques can be considered (30, 31). An example of minimally invasive techniques is the use of thread lifting (32). This method has been used to restore symmetry, by static suspension of the muscles on the side with facial palsy. The researchers combined static suspension on the affected side to botulin toxin on the healthy side. The combination of two techniques improved facial palsy both in static and in dynamic conditions; in fact, all patients improved Sunnybrook scores (37.4 at the baseline vs. 83.3 post treatment) and dynamic facial asymmetry ratios (0.58 at the baseline vs. 0.92 after treatment). Overall, 82.4% patients were satisfied with treatment (31).

Ozturan et al. in 2023 proposed the use of sutureless transconjunctival insertion of an eyelid gold weight to treat lagophthalmos in patients with facial palsy (33). The study was conducted on only six patients, who were followed for 6 months after treatment. The authors reported satisfactory results without patient discomfort or need for re-operation and concluded that their technique is practical, relatively easy and fast to perform. The preservation of the attachment of the elevator muscle to the tarsus allows the surgeon to obtain results like conventional methods. Sutureless method reduce the need for external wound care, burden of suture removal, and suture related complication, such as extrusion (31).

Our proposed technique could potentially be used in association with thread lifting, and it can be modified following Ozturan suggestions and performed with transconjunctival approach to reduce the risk related to the external suture (extrusion, infection, and patient’s discomfort). The main advantage of our lower eyelid surgery (18) is that it can be modified (tailoring muscular flap sutures) depending on the patient’s need and does not present the typical risks observed in case of gold weight or other weight used to support the closure of the superior eyelid (14–17). Moreover, the lower eyelid surgery treats the problem at the site of impairment (lower eyelid) and not indirectly as done by superior eyelid weight.

The combination between physical rehabilitation and minimal eyelid surgery, seems to be, based on our results a good combination to limit the onset of synkinesis, to improve the quality of recovery and to short the recovery times. A small temporizing measure, which can be done in office, may alleviate the difficulties of the patients whom suffer from synkinesis, including drinking and eating concerns.

**Figure 4:**
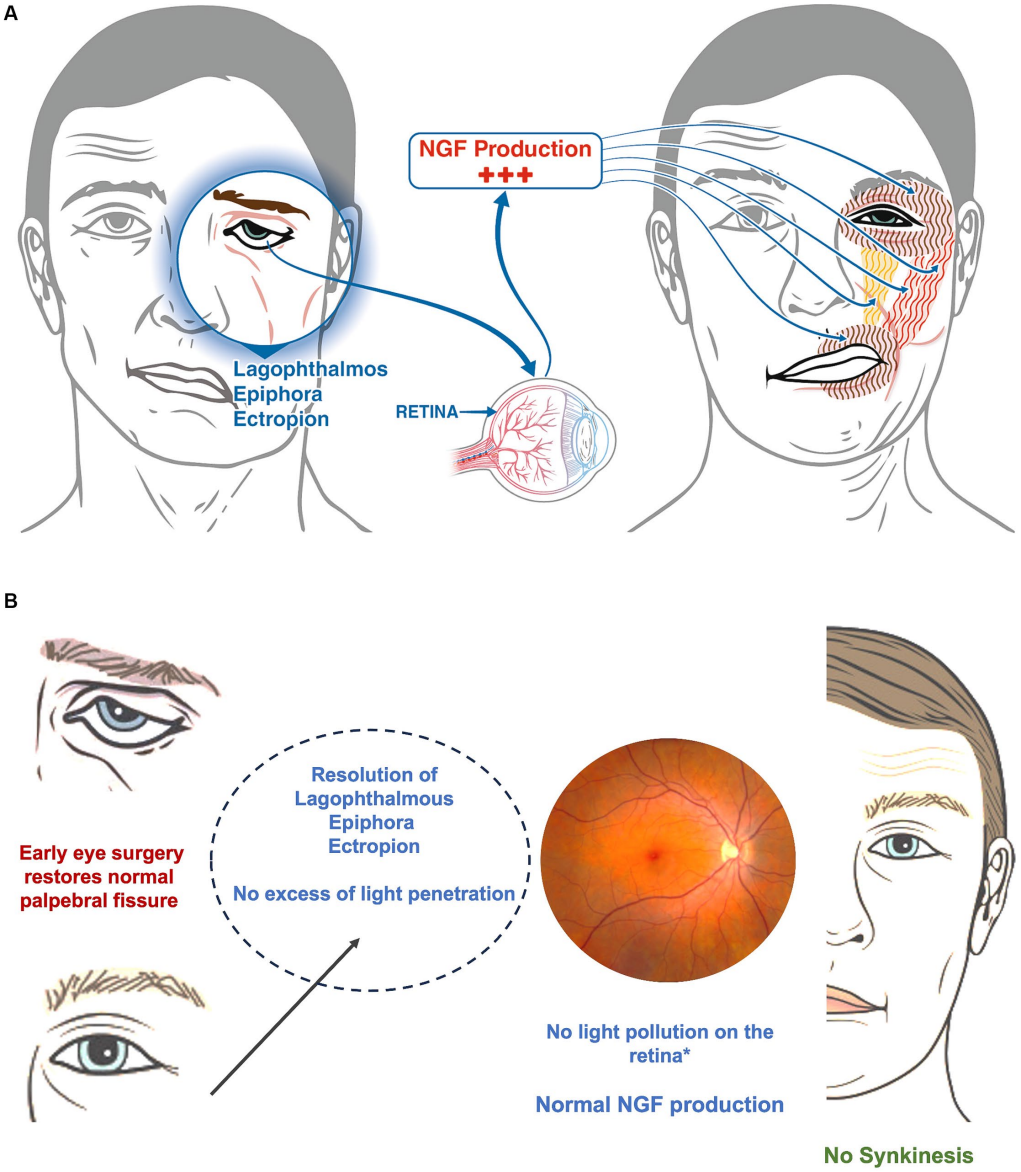
**(A)** In a patient with a facial palsy, the incapacity to close the eye causes both eye dryness and hyper stimulation of the retina because of an excess of light. The retina hyper produces the NGF that stimulate aberrant growth of the facial nerve (not illustrated in the image) with hyper stimulation of the orbicular muscles (eye and mouth-brown vertical line), the major and minor zygomatic muscles (red vertical lines) and the superior elevator lip muscle (yellow). **(B)** The image shows what could happen once soft eye surgery is performed. The resolution of the lagophthalmos and the dryness of the eye, as well as the reduction of the excess of light on the retina (called light pollution) could stop the hyperproduction of NGF avoiding the onset of synkinesis.

The eye is consider the window to the brain (34), it is well known that ocular disorders often precede onset of full clinical manifestations neurological conditions, as in the optic neuritis and internuclear ophthalmoplegia of Multiple Sclerosis (MS). Based on this concept, it might be possible that in presence of excessive dryness of the eye and light overexposure (6), the retina, perhaps even under brain influence, starts to hyperproduce nerve growth factor (NGF) (7). NGF is crucial for ocular surface moisture, corneal integrity, and overall eye health (35, 36) (Figure 4A), and might be responsible of aberrant neuronal regeneration and reinnervation (2) on the base of synkinesis. The latter general couples of the orbicular muscles (eye and mouth) through the action of major and minor zygomatic muscles and superior elevator lip muscle (2) (Figure 4). Excess light penetration (37, 38) due to inadequate eye closure or absence of natural blinking, could induce over production of NGF, promoting aberrant facial nerve regeneration (38). The absence of synkinesis in patients undergoing early lower eyelid surgery might be attributable to the procedure’s pre-emptive protection of the eye, inhibiting retinal overproduction of NGF and thus facilitating normal facial function restoration.

The eyelid surgery described herein, aimed at correcting lagophthalmos with ectropion or epiphora by supporting the eyelid, mitigates conjunctival irritation and eyestrain, potentially reducing the risk of developing synkinesis (Figure 4B). Furthermore, early eye closure provided by the surgery might prevent compensatory brain mechanisms typically leading to synkinesis (39–42) such as exaggerated compensation originating in the contralateral insula (40) and gray matter remodeling ipsilaterally (42). While animal studies, such as those on primates, are necessary to confirm the link between retinal NGF production and aberrant facial nerve regeneration, our findings align with the recognized effectiveness of Kabat therapy in restoring facial movements. Importantly, since eye closure significantly influences ADS scores, surgical support in this area could notably impact both the overall rehabilitation outcomes and the recovery timeline, as demonstrated by the significant differences between the two study groups (Figures 1, 2). Further studies are needed to corroborate findings of the present study and to better understand associated mechanisms.

### Future directions

The results of this study, provide an impetus for further investigation into the role of the eye in the regrowth of damaged facial nerves. The exposure of the sclera induces dryness that might stimulate hyperproduction of NGF and consequent synkinesis onset; on the other hand, over exposure of the retina to the light can also induce hyperproduction of NGF.

Early lower eyelid surgery could be valuable for patients with high risk of non-recovery, as for example aging patients or individuals who are post-stroke. Moreover, additional studies could explore the role for eye drops able to hydrate and create a film on the cornea to protect retina.

### Study limitations

The study has several limitations including the small sample size, limited outcome measures, and heterogeneity, despite similarity in baseline severity of facial nerve impairment between groups. The small sample size precluded age-stratification or analysis of other covariates; elderly patients could be more at risk of epiphora and ectropion due to physiological laxity of eyelid or may differ in regenerative capacity and patterns of recovery. We did not perform electromyography because of limited access to this test. This electrophysiologic test is important to determine the severity of neuronal damage. The study also did not include an arm without facial nerve therapy, nor did it include other potential interventions. Due to the nature of the interventions, blinding of investigators was not feasible, which may introduce bias in outcome assessment. The findings should therefore be regarded as preliminary, and future studies with larger enrolment allowing for detailed analyses by etiology of facial paralysis are needed. Finally, the mechanistic explanation about the protective effect of the early eye surgery is only speculative and so animal studies to confirm the hypothesis are mandatory. However, these clinical results can be useful as preliminary data for further, even translational, studies.

## Conclusion

Early intervention with conservative eyelid surgery to correct lagophthalmos and epiphora may have a role in promoting favorable outcomes after facial paralysis. In the present study, patients undergoing eyelid surgery combined with facial nerve therapy had swifter recovery and lower risk of synkinesis than patients receiving facial therapy alone. Physical rehabilitation remains a proven effective method for restoring normal facial motility and aiding functional recovery. Integrating eye surgery can potentially mitigate the development of synkinesis and expedite recovery, facilitating a quicker return to daily activities for patients. This minimally invasive procedure can be performed under local anesthesia, providing potential benefits with minimal intervention. However, while our findings are promising, they should be regarded as preliminary due to sample size and outcomes assessed. Future studies with larger sample sizes, diverse patient populations, and other therapies are needed to corroborate these preliminary findings and explore underlying mechanisms.

## Data Availability

The raw data supporting the conclusions of this article will be made available by the authors, without undue reservation.
